# Clinical Notes from the McKillip Veterinary College, Chicago, Ill.

**Published:** 1898-06

**Authors:** 


### CLINICAL NOTES FROM THE McKILLIP VETERINARY COLLEGE, CHICAGO, ILL.

Staphylotomy. It was demonstrated at a surgical clinic at this college that the guttural pouches containing pus are best reached through the fauces.

The patient was placed in the dorsal position, and the hand containing a guarded bistoury was passed through the fauces into the pharynx, and an incision made in the median line. The pouches, containing two pints of inspissated pus, were emptied and the patien —a large draught horse—made an immediate and lasting recovery.

A tracheotomy tube was inserted to prevent asphyxiation and as a means of flushing the upper air-passages after the operation.

MALLEIN. The conclusions of Prof. Nocard as to the therapeutic value of mallein were confirmed by a series of experiments on four hundred glandered horses by Dr. J. M. Wright, of this college. Outdoor exercise, healthful surroundings, and periodical injections of mallein repeatedly cured cases not clinically developed.

